# Fetal sex and maternal pregnancy outcomes: a systematic review and meta-analysis

**DOI:** 10.1186/s13293-020-00299-3

**Published:** 2020-05-11

**Authors:** Zoe A. Broere-Brown, Maria C. Adank, Laura Benschop, Myrte Tielemans, Taulant Muka, Romy Gonçalves, Wichor M. Bramer, Josje D Schoufour, Trudy Voortman, Eric A. P. Steegers, Oscar H. Franco, Sarah Schalekamp-Timmermans

**Affiliations:** 1 Department of Obstetrics and Gynecology, Erasmus Medical Center, Rotterdam, the Netherlands; 2 Generation R Study Group, Erasmus Medical Center, Rotterdam, the Netherlands; 3 Department of Epidemiology, Erasmus Medical Center, Rotterdam, the Netherlands; 4 Department Microbiology and Infectious Diseases, Erasmus Medical Center, Rotterdam, the Netherlands; 5 Institute of Social and Preventive Medicine, University of Bern, Bern, Switzerland; 6 Medical Library, Erasmus Medical Center, Rotterdam, the Netherlands; 7 Hogeschool van Amsterdam (HvA), Amsterdam, the Netherlands

**Keywords:** Fetal sex, Pregnancy complications

## Abstract

**Background:**

Since the placenta also has a sex, fetal sex–specific differences in the occurrence of placenta-mediated complications could exist.

**Objective:**

To determine the association of fetal sex with multiple maternal pregnancy complications.

**Search strategy:**

Six electronic databases Ovid MEDLINE, EMBASE, Cochrane Central, Web-of-Science, PubMed, and Google Scholar were systematically searched to identify eligible studies. Reference lists of the included studies and contact with experts were also used for identification of studies.

**Selection criteria:**

Observational studies that assessed fetal sex and the presence of maternal pregnancy complications within singleton pregnancies.

**Data collection and analyses:**

Data were extracted by 2 independent reviewers using a predesigned data collection form.

**Main results:**

From 6522 original references, 74 studies were selected, including over 12,5 million women. Male fetal sex was associated with term pre-eclampsia (pooled OR 1.07 [95%CI 1.06 to 1.09]) and gestational diabetes (pooled OR 1.04 [1.02 to 1.07]). All other pregnancy complications (i.e., gestational hypertension, total pre-eclampsia, eclampsia, placental abruption, and post-partum hemorrhage) tended to be associated with male fetal sex, except for preterm pre-eclampsia, which was more associated with female fetal sex. Overall quality of the included studies was good. Between-study heterogeneity was high due to differences in study population and outcome definition.

**Conclusion:**

This meta-analysis suggests that the occurrence of pregnancy complications differ according to fetal sex with a higher cardiovascular and metabolic load for the mother in the presence of a male fetus.

**Funding:**

None.

## Introduction

In pregnancy, the placenta constitutes the active interface between the maternal and fetal blood circulation. It regulates important physiological changes during pregnancy and accounts for fetal development and nutrient supply. Maternal physiological changes include cardiovascular changes in vascular tone, cardiac output, and plasma volume, providing a better placental perfusion [1, 2]. Impaired placentation leading to abnormal placental perfusion and hence placental dysfunction is believed to be the foundation of several pregnancy complications such as pre-eclampsia [3, 4]. The central role of the placenta in maternal health suggests an intensive interplay between the mother and the placenta, which might be sex dependent. During pregnancy, clear fetal sex-specific differences are noticeable in the occurrence of different pregnancy complications such as pre-eclampsia and gestational diabetes and even in maternal vascular adaptation to pregnancy [5]. Despite growing speculations that placentation and maternal adaptation to pregnancy are influenced by fetal sex, in most studies that assess these possible pathophysiological mechanisms, fetal sex is not being taken into account [6–9].

Several systematic reviews and meta-analyses have been performed to investigate the association between fetal sex and single pregnancy complications such as pre-eclampsia or gestational diabetes. It is plausible that if fetal sex is associated with one maternal pregnancy complication it might be associated with other pregnancy complications as well. However, some of the performed systematic reviews had restrictions concerning publication date and source population and did not check the references for additional inclusions. We conducted a systematic review and meta-analysis of observational studies evaluating the association of fetal sex with multiple maternal pregnancy complications. To explore the worldwide impact of fetal sex on these maternal pregnancy complications, population attributable factors (PAF) were calculated.

## Materials and methods

### Data sources and search strategy

This review was conducted using a predefined protocol and in accordance with PRISMA and MOOSE guidelines (Additional file 3 and Additional file 4) [10, 11]. Six electronic databases (Ovid MEDLINE, EMBASE, Cochrane Central, Web-of-Science, PubMed, and Google Scholar) were searched until April 5, 2019, without language or publication date restriction. The computer-based searches combined terms related to (1) the exposure such as (gender, sex, fetus, embryo, and baby); (2) maternal pregnancy complications (e.g., gestational hypertension, pre-eclampsia (total, preterm, term, and postterm), eclampsia, gestational diabetes, placental abruption, post-partum hemorrhage, and miscarriage); and (3) relevant population (humans, singleton pregnancy) (Additional file 1). Two independent reviewers screened the titles and abstracts of all studies initially identified, according to the selection criteria. Any disagreement was resolved through consensus or consultation with a third independent reviewer. Full texts were retrieved from studies that satisfied all selection criteria. From each selected manuscript we also searched their individual reference list for other possible includable studies. For this, we used a restriction of 20% most recently published studies.

### Study selection and eligibility criteria

Observational studies were eligible if they assessed fetal sex as primary exposure in singleton pregnancies and collected end points for maternal pregnancy complications, including gestational hypertension, pre-eclampsia, eclampsia, gestational diabetes, placental abruption, post-partum hemorrhage, and miscarriage. Study populations in the eligible studies included women recruited from health care settings or general populations. Studies on newborns with an abnormal karyogram, congenital conditions involving sex steroids and/or sex characteristics were excluded.

### Data extraction

Two authors independently extracted data and consensus was reached in case of any inconsistency with involvement of a third author. A predesigned electronic data extraction form was used to collect relevant information. The data collection form included questions on qualitative aspects of the study (such as date of publication, design, geographical origin and setting, funding source, selection criteria, patient samplings, and location of research group), participant characteristics (such as number included in the analysis, age, ethnicity, comorbidities) and information on the reported outcome (type of outcome, outcome assessment method, statistical analysis, adjustment variables). In instances of multiple publications, the most up-to-date and comprehensive information was extracted.

### Assessing study quality

Two reviewers independently rated the quality of studies using the Newcastle–Ottawa Quality Assessment Scale (Additional file 2). This quality score system is applicable for case-control and cohort studies. The system allocates points for information on participants, comparability, and outcome with a maximum of eight points.

### Statistical Analysis

We evaluated the differences between pregnancies with a male and female fetus on maternal pregnancy complications (including gestational hypertension, pre-eclampsia (total, preterm, term, and postterm), eclampsia, gestational diabetes, placental abruption, post-partum hemorrhage, and miscarriage). To enable a consistent approach to the meta-analysis and enhance interpretation of the findings, effect estimates were converted where appropriate. The inverse variance weighted method was used to combine summary measures using random-effects models to minimize effects of between-study heterogeneity [12]. The summary estimates presented were calculated using random-effects models (D+L) and fixed effects (I+V). We also conducted sensitivity analyses using fixed-effects models. Heterogeneity was assessed using the Cochrane _*X*_^2^ statistic and the *I*^2^ statistic and was distinguished as low (*I*^2^ ≤ 25%), moderate (*I*^2^ > 25% and < 75%), or high (*I*^2^ ≥ 75%) [13].

Sensitivity analyses were performed by restricting the analysis to studies with very strict in- or exclusion criteria resulting in a specific participant population (e.g., only inclusion of nulligravid, or women who were admitted with hyperemesis gravidarum patients or had gestational diabetes/placental abruption/SGA, etc.). Stratified analyses were performed on geographical location (Western vs non-Western), number of participants (< 10.000 vs ≥ 10.000), study design (case-control vs retrospective cohorts vs prospective cohort), and on quality score (< 7 vs ≥ 7), which were pre-specified as characteristics of assessment of heterogeneity and, in addition to stratification, were evaluated using random-effects meta-regression. Population attributable fractions (PAF) were calculated as PAF = (*p* (RR − 1))/(*p* (RR – 1) + 1) [14]. The PAF is an epidemiological measure widely used to assess the public health impact of exposures in a population. It describes the proportional reduction in population disease or mortality that would occur if the exposure to a risk factor was reduced to an alternative ideal exposure scenario (i.e., female fetal sex). A narrative synthesis and construction of descriptive summary tables were performed for these studies that could not be quantitatively pooled.

All tests were 2-tailed; *p* ≤ 0.05 was considered statistically significant. Stata release 13 (StataCorp) was used for all analyses.

## Results

### Study identification and selection

**Fig. 1:**
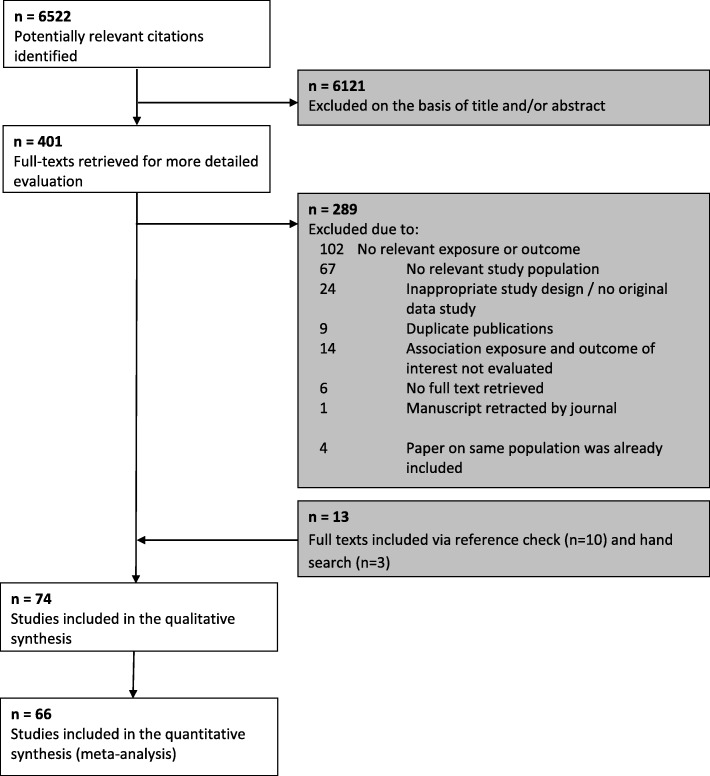
Search strategy for the studies included in the current systematic review (search until April 5, 2019). PRISMA flow diagram of selection process of eligible studies

We identified 6522 relevant citations. After screening titles and abstracts, 401 articles were selected for detailed evaluation of their full texts. Of those, 74 articles met our inclusion criteria and were included in the review (Table 1, Fig. 1).

**Table 1:** Associations between fetal sex and maternal pregnancy outcomes

First author	Statistical analyses	Subgroups	Tendency towards which sex (M/F/=)	Crude effect estimate (95% CI)	*p* value	Covariate adjustment	Adjusted effect estimate (95% CI)	*p* value
Gestational hypertension								
Andersen et al. 2016 [15]	Logistic regression		F	0..69 (0..38–1..25)	0.22			
Baibergenova et al. 2006 [16]	Logistic regression		F	1.06 (0.55–2.50)	0.87			
Campbell et al. 1983 [17]	Logistic regression		M	1.18 (1.09–1.27)	< 0.0001			
Chien et al. 2011 [18]	Logistic regression		M	0.97 (0.96–0.98)	< 0.0001			
Engel et al. 2008 [19]	Chi-square	Total	M	1.04 (0.94–1.14)	0.46			
		Mild	M	1.04 (0.94–1.16)	0.44			
		Moderate	F	0.99 (0.80–1.24)	0.95			
		Severe	F	0.94 (0.62–1.42)	0.76			
Favilli et al. 2013 [20]	Logistic regression		F	1.69 (0.63–4.57)	0.43	Maternal age > 40 years, weight gain, BMI, gestational diabetes	0.98 (0.43–2.25)	0.97
Hou et al. 2014 [21]	Logistic regression		F	0.97 (0.91–1.02)	0.25			
Juberg et al. 1976 [22]	Chi-square		M		0.03			
Li et al. 2016 [23]	Logistic regression		F	0.97 (0.78–1.21)	0.79			
Makhseed et al. 1998 [24]	Logistic regression	Total	M	1.01 (0.86–1.20)	0.87			
		Primiparous	F	0.87 (0.65–1.17)	0.36			
		Multiparous	M	1.09 (0.89–1.33)	0.42			
Persson et al. 2014 [25]	Logistic regression	Healthy population	M	1.03 (1.01–1.06)	0.003			
		Gestational diabetes	M	1.08 (0.93–1.26)	0.31			
		Diabetes mellitus type I	F	0.93 (0.79–1.09)	0.35			
		Diabetes mellitus type II	F	0.83 (0.44–1.57)	0.56			
Ricart et al. 2009 [76]	Logistic regression		M	1.22 (0.91–1.63)	0.19			
Sheiner et al. 2004 [26]	Logistic regression		=	1.00 (0.95–1.05)	0.96			
Shiozaki et al. 2011 [27]	Logistic regression		F	0.88 (0.83–0.92)	< 0.0001			
Sykes et al. 2014 [77]	Logistic regression		M	1.33 (0.67–2.63)	0.42			
Tundidor et al. 2012 [28]	Relative risk		F	0.81 (0.55–1.20)	NR			
Valvi et al. 2017 [109]	Logistic regression		M	1.03 (0.58–1.85)	0.91			
Verburg et al. 2016 [29]	Relative risk	Total	M	1.05 (1.03–1.07)	NR			
		25–29 weeks	F	0.69 (0.58–0.81)	NR			
		30–33 weeks	F	0.87 (0.79–0.97)	NR			
		34–36 weeks	F	0.93 (0.87–0.98)	NR			
		37–39 weeks	M	1.06 (1.04–1.09)	NR			
		40–42 weeks	M	1.07 (1.04–1.11)	NR			
Zheng et al. 2016 [30]	Logistic regression		F	0.54 (0.26–1.14)	0.11			
Pre-eclampsia								
Aibar et al. 2012 [31]	Logistic regression		F	0.99 (0.65–1.49)	0.94			
Aliyu et al. 2012 [32]	Logistic regression		F	0.90 (0.79–1.03)	0.12			
Andersen et al. 2016 [15]	Logistic regression	Total	F	0.95 (0.69–1.31)	0.76			
		Preterm	F	1.04 (0.42–2.56)	0.94			
		Term	M	1.22 (0.85–1.74)	0.29			
Basso et al. 2001 [33]	Logistic regression		M	0.94 (0.92–0.97)	< 0.05			
Brettel et al. 2008 [34]	Logistic regression		F	1.17 (1.01–1.35)	0.03			
Campbell et al. 1983 [17]	Logistic regression		F	1.08 (0.94–1.24)	0.3			
Choong et al. 1995 [35]	Logistic regression		F	1.45 (1.22–1.71)	< 0.0001			
Chu et al. 2014 [36]	Logistic regression		M	0.60 (0.19–1.83)	0.39			
Hadar et al. 2017 [37]	Logistic regression		F	0.99 (0.68–1.43)	0.95			
Hou et al. 2014 [21]	Logistic regression		F	0.95 (0.88–1.02)	0.13			
Juberg et al. 1976 [22]	Chi-square		M		0.06			
Khalil et al. 2013 [38]	Logistic regression	Total	M	1.04 (0.91–1.19)	0.57			
		Preterm	F	1.53 (1.07–2.20)	0.02			
		Term	M	1.08 (0.93–1.25)	0.31			
		Postterm	M	3.46 (1.40–8.53)	0.007			
Lao et al. 2011 [39]	Logistic regression		F	0.92 (0.81–1.06)	0.26			
Lao et al. 2017 [40]	Logistic regression		M	1.56 (1.41–1.73)	<0.0001			
Li et al. 2016 [23]	Logistic regression		F	0.66 (0.45–0.98)	0.04			
Lisonkova et al. 2013 [41]	Cox regression	< 34 weeks	M	1.10 (1.07–1.14)	NR	NR	1.10 (1.06–1.14)	NR
		> 34 weeks	M	1.10 (1.07–1.14)	NR	NR	1.10 (1.06–1.14)	NR
Liu et al. 2016 [42]	Logistic regression	Total		0.96 (0.88–1.04)	0.31			
		Preterm		1.15 (1.00–1.32)	0.046			
Makhseed et al. 1998 [24]	Logistic regression	Total	F	0.92 (0.68–1.24)	0.57			
		Nulliparous	F	0.74 (0.49–1.10)	0.13			
		Multiparous	M	1.20 (0.76–1.90)	0.43			
Masoumi et al. 2017 [43]	Logistic regression	Total	M	1.09 (0.90–1.31)	0.40			
		Severe	M	1.43 (0.81–2.51)	0.21			
Morsing et al. 2018 [44]	Logistic regression		F	0.80 (0.59–1.09)	0.16			
Myers et al. 2015 [45]	Logistic regression		=	0.94 (0.65–1.36)	0.74			
Peled et al. 2013 [46]	Logistic regression		M	1.79 (0.42–7.56)	0.43			
Persson et al. 2014 [25]	Logistic regression	Healthy population	M	1.03 (1.01–1.06)	0.003			
		Gestational diabetes	M	1.08 (0.93–1.26)	0.31			
		Diabetes mellitus type I	F	0.93 (0.79–1.09)	0.35			
		Diabetes mellitus type II	F	0.83 (0.44–1.57)	0.56			
Quiñones et al. 2005 [47]	Logistic regression		M	1.15 (0.77–1.70)	0.5			
Reynolds et al. 2012 [48]	Logistic regression	Total	F	0.85 (0.71–1.02)	0.08			
		Preterm	F	1.25 (0.79–1.97)	0.34			
		Term	F	0.86 (0.71–1.04)	0.13			
Roy et al. 2015 [49]	Logistic regression	Total	M	1.28 (0.72–2.29)	0.4			
		Preterm	M	0.77 (0.33–1.81)	0.55			
		Term	M	1.28 (0.66–2.46)	0.46			
Sharifzadeh et al. 2012 [50]			F	0.88 (0.33–2.35)	0.8			
Sheiner et al. 2004 [26]	Logistic regression		=	1.00 (0.95–1.05)	0.96			
Shiozaki et al. 2011 [27]	Chi-square	Pre-eclampsia	F	0.84 (0.79–0.89)	< 0.001			
		Pre-eclampsia with fetal death	M	1.21 (0.70–1.48)	0.95			
		Severe pre-eclampsia	F	1.21 (1.10–1.33)	0.001			
		Severe pre-eclampsia with fetal death	F	1.14 (0.67–1.93)	0.63			
Sykes et al. 2014 [77]	Logistic regression		M	1.27 (0.64–2.51)	0.49			
Taylor et al. 2018 [51]	Logistic regression		F	0.94 (0.67–1.30)	0.70			
Taylor et al. 2018 [51]	Logistic regression	PE overall	F	0.89 (0.64–1.24)	0.69			
		Term (> 37 weeks)	F	0.92 (0.65–1.30)	0.63			
		Preterm (<37 weeks)	F	0.72 (0.37–1.39)	0.32			
		Very preterm (<34 weeks)	F	0.38 (0.13–1.07)	0.07			
Toivanen et al. 1970 [52]	Logistic regression		M	1.20 (1.06–1.37)	0.005			
Trudel et al. 2015 [53]	Logistic regression		M	1.01 (0.95–1.07)	0.82			
Vatten et al. 2004 [54]	Logistic regression	Total	M	1.05 (1.03–1.07)	< 0.0001			
		Preterm (< 37 weeks)	F	1.17 (1.11–1.22)	< 0.0001			
		Term (37–42 weeks)	M	1.06 (1.04–1.08	< 0.0001			
		Postterm (> 42 weeks)	M	1.07 (0.96–1.18)	0.23			
		25–29 weeks	F	1.55 (1.31–1.83)	< 0.0001			
		30–33 weeks	F	1.33 (1.21–1.46)	< 0.0001			
		34–36 wls	F	1.07 (1.01–1.14)	0.03			
		37–39 weeks	F	0.98 (0.85–1.01)	0.18			
		40–42 weeks	M	1.10 (1.07–1.13)	< 0.0001			
Verburg et al. 2016 [29]	Relative risk	Total	M	1.05 (1.03–1.07)	NR			
		25–29 weeks	F	0.69 (0.58–0.81)	NR			
		30–33 weeks	F	0.87 (0.79–0.97)	NR			
		34–36 weeks	F	0.93 (0.87–0.98)	NR			
		37–39 weeks	M	1.06 (1.04–1.09)	NR			
		40–42 weeks	M	1.07 (1.04–1.11)	NR			
Wandabwa et al. 2010 [79]	Logistic regression		F	0.65 (0.45–0.95)	0.03			
Weinberg et al. 2017 [55]	Logistic regression	Total	M	1.01 (0.98–1.04)	0.71			
		Term (> 37 weeks)	M	1.05 (1.01–1.08)	0.01			
		Preterm (<37 weeks)	F	0.89 (0.84–0.94)	0.0001			
Zheng et al. 2016 [30]	Logistic regression	Total	F	0.49 (0.27–0.89)	0.02			
		Mild	F	0.65 (0.30–1.43)	0.29			
		Severe	F	2.60 (1.18–5.73)	0.02			
Eclampsia								
Aibar et al. 2012 [31]	Logistic regression		M	1.54 (0.50–4.72)	0.45			
Aliyu et al. 2012 [32]	Logistic regression		F	0.92 (0.42–2.01)	0.83			
Campbell et al. 1983 [17]	Logistic regression		F	0.89 (0.35–2.32)	0.82			
Chien et al. 2011 [18]	Logistic regression		=	1.00 (0.97–1.04)	0.89			
Hou et al. 2014 [21]	Chi-square		M		0.13			
Llopez-Lera et al. 1990 [82]	Chi-square		M		< 0.05			
Persson et al. 2014 [25]	Logistic regression	Healthy population	M	1.03 (1.01–1.06)	0.003			
		Gestational diabetes	M	1.08 (0.93–1.26)	0.31			
		Diabetes mellitus type I	F	0.93 (0.79–1.09)	0.35			
		Diabetes mellitus type II	F	0.83 (0.44–1.57)	0.56			
Wandabwa et al. 2010 [79]	Logistic regression		F	0.65 (0.45–0.95)	0.03			
Gestational diabetes								
Aibar et al. 2012 [31]	Logistic regression		M	1.21 (1.06–1.37)	0.0034			
Breschi et al. 1993 [56]	Logistic regression		F	0.96 (0.36–2.52)	0.93			
Cosson et al. 2016 [57]	Logistic regression		=	1.00 (0.93–1.08)	0.96			
Ehrlich et al. 2012 [58]	Logistic regression		M	1.02 (0.99–1.05)	NR	Maternal ethnicity	1.02 (0.99–1.05)	NR
						Maternal ethnicity. education and age	1.02 (0.99–1.05)	NR
Engel et al. 2008 [19]	Logistic regression		M	1.07 (0.85–1.36)	0.54			
Favili et al. 2013 [20]	Logistic regression		M	2.36 (0.58–9.61)	0.37	Maternal age > 40 years, BMI, weight gain, gestational hypertension	0.95 (0.37–2.46)	0.92
Heckbert et al. 1988 [59]	Logistic regression		F	0.97 (0.77–1.21)	0.79			
Hou et al. 2014 [21]	Logistic regression		M	1.01 (0.96–1.07)	0.61			
Janssen et al. 1996 [60]	Logistic regression		M	1.02 (0.96–1.08)	0.5			
Kale et al. 2005 [61]	Logistic regression		M	1.64 (1.12–2.40)	0.01			
Khalil et al. 2013 [38]	Logistic regression		M	1.41 (1.15–1.72)	< 0.001			
Lao et al. 2011 [39]	Logistic regression		M	1.05 (0.99–1.12	0.12			
Lao et al. 2017 [40]	Logistic regression		M	1.06 (1.01–1.11)	0.08			
Lawlor et al. 2009 [84]	Logistic regression		M	1.61 (0.92–2.81)	0.09			
Liu et al. 2016 [42]	Logistic regression		M	1.08 (1.00–1.16)	0.048			
Macaulay et al. 2018 [86]	Logistic regression		M	1.16 (0.73–1.84)	0.53			
Oken et al. 2016 [62]	Logistic regression		M	1.39 (0.81–2.36)	0.23			
Okereke et al. 2002 [63]	Logistic regression		M	1.39 (0.81–2.36)	0.23			
Peled et al. 2013 [46]	Logistic regression		M	3.24 (0.65–16.22)	0.15			
Retnakaran et al. 2015 [64]	Logistic regression		M	1.03 (1.00–1.05)	0.047			
Retnakaran et al. 2015 [64]	Logistic regression		M	1.24 (0.92–1.67)	0.16			
Ricart et al. 2009 [76]	Logistic regression		M	1.05 (0.91–1.22)	0.17			
Sheiner et al. 2004 [26]	Logistic regression		M	1.07 (1.01–1.12)	0.01			
Spellacy et al. 1985 [65]	Chi-square		M		NS			
Strutz et al. 2018 [66]	Logistic regression		M	1.80 (0.40–8.18)	0.45			
Trudel et al. 2015 [53]	Logistic regression		F	0.96 (0.90–1.04)	0.32			
Verburg et al. 2016 [29]	RR		M	1.04 (1.01–1.07)	NR			
Xiao et al. 2014 [67]	Logistic regression		M	1.29 (0.58–2.89)	0.53			
Placental abruption								
Aliyu et al. 2012 [32]	Logistic regression		F	0.98 (0.87–1.12)	0.8			
Brettel et al. 2008 [34]	Logistic regression		M	1.29 (0.97–1.71)	0.08			
Engel et al. 2008 [19]	Logistic regression		F	0.53 (0.28–0.99)	0.049			
Hou et al. 2014 [21]	Logistic regression		F	0.98 (0.83–1.15)	0.76			
Jakobovits et al. 1988 [68]	Chi-square	Total	M		NS			
		17–20 years	M		< 0.001			
		21–25 years	M		< 0.01			
		26–30 years	F		NS			
		31–35 years	M		< 0.05			
		36–40 years	M		< 0.05			
		41–42 years	=		NS			
Lopez-Llera et al. 1990 [82]	Logistic regression		M	0.94 (0.54–1.66)	0.84			
Peled et al. 2013 [46]	Logistic regression		M	2.90 (0.76–11.03)	0.12			
Raissanen et al. 2013 [110]	Logistic regression	Total	M	1.19 (1.12–1.26)	< 0.0001			
		Nulliparous	M	1.23 (1.12–1.36)	< 0.0001	NR	1.36 (1.23–1.51)	
		Multiparous	M	1.16 (1.08–1.26)	0.001	NR	1.38 (1.27–1.50)	
Schildberger et al. 2016 [69]	Logistic regression		F	0.84 (0.81–0.87)	< 0.0001			
Sheiner et al. 2002 [70]	Logistic regression		F	0.98 (0.78–1.24)	0.88			
Sheiner et al. 2004 [26]	Logistic regression		M	1.15 (0.89–1.49)	0.28			
Tikkanen et al. 2013 [90]	Logistic regression		M	1.18 (1.11–1.25)	< 0.0001			
Wandabwa et al. 2005 [91]	Logistic regression		M	2.20 (1.20–4.90)	< 0.01	Distance to hospital. age, type of house, hypertension, previous caesarean section, previous stillbirth	1.90 (1.00–3.80)	NR
Weissmann–Brenner et al. 2015 [71]	Logistic regression	Total	M	1.20 (0.77–1.87)	0.42			
		Age < 40 years	M	1.14 (0.73–1.79)	0.56			
		Age > 40 years	M	5.08 (0.24–106.0)	0.29			
Post-partum hemorrhage								
Favili et al. 2013 [20]	Logistic regression	Total	M	1.12 (0.34–3.72)	0.85			
		Age ≥ 40 years	M	2.10 (0.40–11.01)	0.38			
		Age < 40 years	F	0.35 (0.04–3.37)	0.36			
Weissmann–Brenner et al. 2015 [71]	Logistic regression	Total	M	1.20 (0.88–1.65)	0.25			
		Age ≥ 40 years	M	1.16 (0.84–1.61)	0.35			
		Age < 40 years	M	4.07 (0.45–36.5)	0.21			
Liu et al. 2016 [42]	Logistic regression		F	0.91 (0.83–0.99)	0.0046			
Miscarriage								
Byrne et al. 1987 [72]	Risk ratio	Total	M		< 0.05			
		Morphological normal	M		< 0.05			
		Morphological abnormal	F		> 0.05			
Cheng et al. 2014 [73]	Risk ratio		F		< 0.001			
Del Fabro et al. 2011 [74]	Risk ratio	Total	F		< 0.05			
		4–10 weeks	F		< 0.001			
		11–15 weeks	F		0.07			
		16–20 weeks	F		0.06

### Characteristics of included studies

The 74 included studies reported results for 12.658.554 unique women (Table 1). Forty-seven were retrospective cohort studies, 10 prospective cohort studies and the remaining 17 studies were case-control studies. The majority of studies were performed in Western countries (22 in Northern America, 25 in Europe, three in Australia, and one study in both Europe and Australia). Of the remaining studies, 11 were performed in Asia, nine in the Middle East, and three in Africa. More than one outcome was measured in 23 studies, and for these, the measure of association for each outcome was included in the analysis. One study was written in Spanish, all other studies were in English [75].

### Association of fetal sex with maternal pregnancy outcomes

#### Fetal sex and gestational hypertension

Of the included studies, 19 investigated gestational hypertension with a total of 5.752.185 participants (Tables 1 and 2) [15–30, 76, 77]. Of these studies, five found an association with male fetal sex dominance, one with female fetal sex dominance, and 13 found no association. Four studies stratified their results. One study stratified for severity of gestational hypertension (mild, moderate, and severe) [19]. None of the subgroups were associated with fetal sex. Another study stratified for parity in which no association was found for both primiparous and multiparous women. Persson et al. stratified for comorbidity (gestational diabetes, diabetes mellitus type 1 or 2) [25]. They observed male fetal sex dominance for gestational hypertension in the non-diabetic group. No such association was found for women with diabetes. The last study stratified for gestational age and found that gestational hypertension was associated with male fetal sex only in term and postterm pregnancies [29].

**Table 2:** Pooled odds ratios of the occurrence of maternal pregnancy complications by study characteristics

Subgroup	No. of studies	Participants	OR (95% CI)	***p*** value for heterogeneity
**Gestational hypertension**				
Geographical location				
Western	11	5.511.340	1.02 (0.98;1.06)	0.3
Non-Western	5	125.016	0.99 (0.95;1.02)	
No of participants				
< 10.000	8	30.853	1.01 (0.98;1.05)	0.47
≥ 10.000	8	5.605.503	0.96 (0.85;1.10)	
Study design				
Case-control	1	294	0.54 (0.26;1.14)	0.19
Retrospective cohort	11	5.508.737	1.02 (0.98;1.05)	
Prospective cohort	4	127.325	0.98 (0.89;1.08)	
Quality score				
< 7	11	5.489.916	1.03 (1.01;1.05)	< 0.001
≥ 7	5	146.440	0.92 (0.81;1.05)	
**Pre-eclampsia (total)**				
Geographical location				
Western	15	3.472.444	1.03 (1.00;1.05)	< 0.001
Non-Western	14	541.647	0.90 (0.83;0.97)	
No. of participants				
< 10.000	13	39.373	0.92 (0.78;1.08)	0.84
≥ 10.000	16	3.974.718	0.97 (0.94;1.01)	
Study design				
Case-control	7	2.174	0.86 (0.64;1.16)	0.12
Retrospective cohort	18	3.884.545	0.98 (0.95;1.02)	
Prospective cohort	4	127.372	0.90 (0.81;1.00)	
Quality score				
< 7	22	1.538.622	0.97 (0.93;1.02)	0.71
≥ 7	7	2.475.469	0.95 (0.88;1.02)	
**Eclampsia**				
Geographical location				
Western	5	4.820.821	1.02 (1.00;1.04)	0.05
Non-Western	2	110.156	0.82 (0.57;1.18)	
No of participants				
< 10.000	1	434	0.65 (0.45;0.94)	0.02
≥ 10.000	6	4.930.534	1.01 (0.99;1.04)	
Study design				
Case-control	1	434	0.65 (0.45;0.95)	0.01
Retrospective cohort	5	4.820.821	0.95 (0.88;1.02)	
Prospective cohort	1	109.722	1.02 (1.00;1.04)	
Quality score				
< 7	6	4.920.963	1.00 (0.95;1.04)	0.84
≥ 7	1	10.014	0.92 (0.42;2.01)	
**Gestational diabetes**				
Geographical location				
Western	16	1.632.560	1.03 (1.01;1.05)	0.17
Non-Western	8	379.756	1.09 (1.02;1.15)	
No of participants				
< 10.000	10	15.111	1.16 (1.02;1.33)	0.14
≥ 10.000	14	1.997.205	1.04 (1.02;1.06)	
Study design				
Case-control	5	1.062	1.15 (0.94;1.40)	0.66
Retrospective cohort	12	2.009.749	1.04 (1.02;1.06)	
Prospective cohort	7	1.505	1.16 (1.01;1.33)	
Quality score				
< 7	18	1.091.263	1.05 (1.02;1.09)	0.75
≥ 7	6	921.053	1.04 (1.01;1.07)	
**Placental abruption**				
Geographical location				
Western	7	2.876.604	1.03 (0.86;1.23)	0.45
Non-Western	6	227.068	1.10 (0.93;1.31)	
No of participants				
< 10.000	4	7.801	1.31 (0.85;2.02)	0.4
≥ 10.000	9	3.095.871	1.04 (0.90;1.22)	
Study design				
Case-control	2	1.090	2.34 (1.25;4.35)	0.08
Retrospective cohort	10	2.992.860	1.05 (0.90;1.22)	
Prospective cohort	1	109.722	0.98 (0.83;1.15)	
Quality score				
< 7	6	224.641	1.21 (0.96;1.51)	0.2
≥ 7	7	2.879.031	1.01 (0.85;1.19)	

**Fig. 2:**
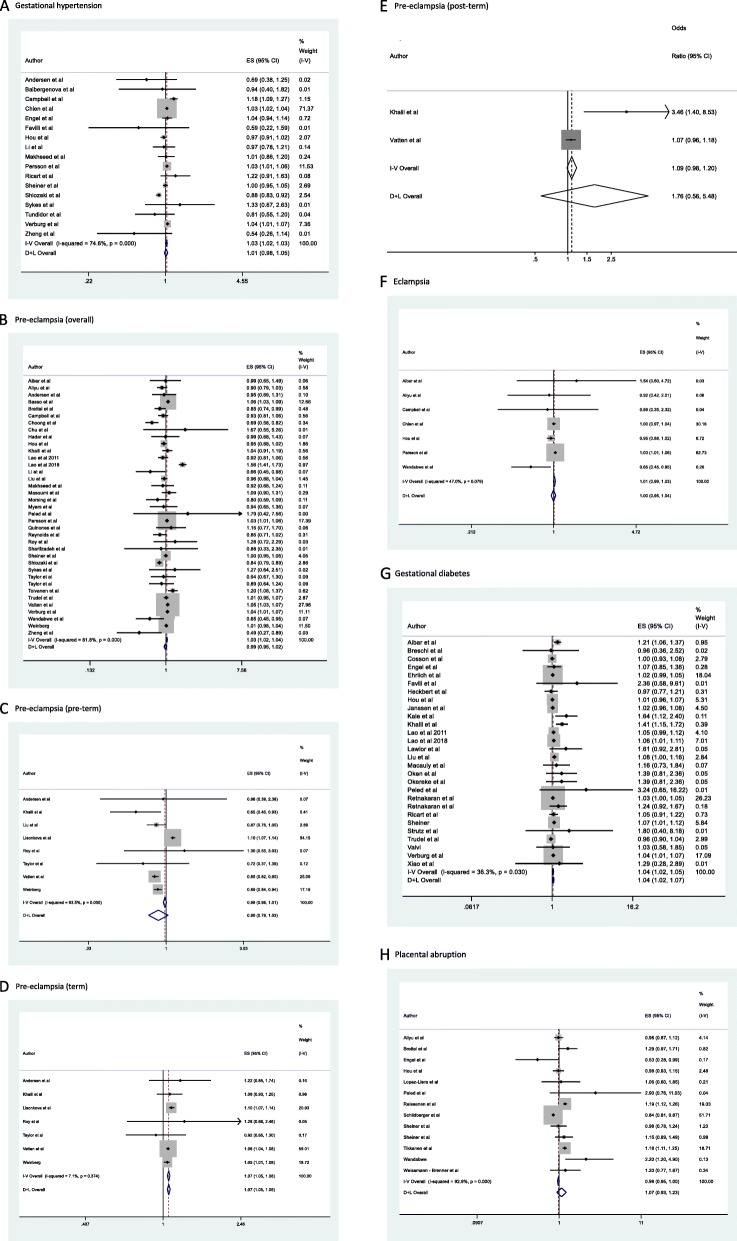
Meta-analyses on the association between fetal sex and maternal pregnancy complications. The boxes are proportional to the weight of each study in the analysis, and the lines represent their 95% confidence intervals (CIs). Size of data markers are proportional to the inverse of the variance of the effect estimate. The open diamond represent the pooled odds ratio, and its width represents its 95% CI. The summary estimates presented were calculated using random-effects models (D + L) and fixed effects (I + V). Assessment of heterogeneity: gestational hypertension (*I*^2^ = 74,8%, *p* < 0.001) (**a**); total pre-eclampsia (*I*^2^ = 81,8%, p < 0.001) (**b**); preterm pre-eclampsia (*I*^2^ = 93,5%, p < 0.001) (**c**); term pre-eclampsia (*I*^2^ = 7,1%, p = 0.37) (**d**); postterm pre-eclampsia (*I*^2^ = 84.4%, *p* = 0.011) (**e**); eclampsia (*I*^2^ = 47.0%, p = 0.08) (**f**); gestational diabetes, (*I*^2^ = 36,3%, p = 0.03) (**g**); placental abruption (*I*^2^ = 92.9%, *p* < 0.001) (**h**)

In our pooled meta-analyses which compared the occurrence of gestational hypertension in women carrying a male fetus compared with women carrying a female fetus, the OR was 1.01 (0.98–1.05) (Fig. 2a). The PAF for total gestational hypertension was 1.31% (95% CI [-0.22;2.84], p = 0.09). Assuming a worldwide prevalence of 7%, this resembles almost 200.000 cases worldwide of gestational hypertension associated to some degree with the presence of a male fetus [78].

#### Fetal sex and pre-eclampsia

Of the included studies, 39 investigated pre-eclampsia with a total of 4.766.334 participants (Tables 1 and 2) [15, 17, 21–27, 29–55, 77, 79, 80]. Eight studies found an association with male fetal sex, six with female fetal sex and the remaining 25 studies did not find a significant association. However, the association between fetal sex and pre-eclampsia was dependent of gestational age. Ten studies stratified their results for gestational age. Two studies stratified their results not only in term vs preterm but additionally investigated several gestational age periods showing a strong association between female pregnancies and very early pre-eclampsia [29, 54]. This association attenuated with gestational age. At term and postterm, the association is reversed and male fetal sex is associated with pre-eclampsia. Three studies stratified into severity of pre-eclampsia [27, 30, 43]. Two of these studies show that a more severe pre-eclampsia is associated with a female fetus while one study shows that severe pre-eclampsia is associated with a male fetus.

In our pooled meta-analyses which compared the occurrence of overall pre-eclampsia (i.e., preterm, term, and postterm) in women carrying a male fetus compared with women carrying a female fetus, the OR was 0.99 (0.95–1.02) (Fig. 2b). For preterm, term, and postterm pre-eclampsia the pooled ORs were 0.90 (0.78–1.03), 1.07 (1.06–1.09) and 1.76 (0.56–5.48) respectively for a male fetus compared to a female fetus (Fig. 2 c, d, and e respectively). The PAF for total pre-eclampsia was 1.23% (95% CI [− 0.64;3.11], *p* = 0.20). Assuming a worldwide prevalence of 5%, this resembles approximately 130.000 cases of pre-eclampsia worldwide associated to some degree with the presence of a female fetus [81].

#### Fetal sex and eclampsia

Of the included studies, eight investigated eclampsia with a total of 4.931.754 participants (Tables 1 and 2) [17, 18, 21, 25, 31, 32, 79, 82]. Two studies found an association with male fetal sex, one study with female fetal sex, the remaining studies did not find a significant association.

In our pooled meta-analyses which compared the occurrence of eclampsia in women carrying a male fetus compared with women carrying a female fetus, the OR was 1.00 (0.95–1.04) (Fig. 2e). The PAF for eclampsia was 0.71% (95% CI [− 3.60;5.02], *p* = 0.75). Assuming a worldwide prevalence of 0.01%, this resembles almost 2000 cases of eclampsia worldwide associated to some degree with the presence of a male fetus [83].

#### Fetal sex and gestational diabetes

Of the included studies, 28 investigated gestational diabetes, with a total of 2.126.446 participants (Tables 1 and 2) [19–21, 26, 29, 31, 38–40, 42, 46, 53, 56–67, 76, 84–86]. Of the included studies seven studies found an association between fetal sex and gestational diabetes all showing a higher rate of gestational diabetes within women carrying a male fetus.

In our pooled meta-analyses which compared the occurrence of gestational diabetes in women carrying a male fetus compared with women carrying a female fetus, the OR was 1.04 (1.02–1.07) (Fig. 2g). The PAF for gestational diabetes was 1.75% (95% CI [1.05;2.46], *p* < 0.001). Assuming a worldwide prevalence of 6%, this resembles almost 225,000 cases of gestational diabetes worldwide associated to some degree with the presence of a male fetus [87].

#### Fetal sex and placental abruption

Of the included studies, 14 investigated placental abruption, with a total of 3.130.530 participants (Tables 1 and 2) [19, 21, 26, 34, 46, 68–71, 82, 88–91]. All studies that found a significant association showed a higher rate of placental abruption within women carrying a male fetus. Two studies stratified their results according to maternal age [68, 71]. Despite stratification, in the majority of age groups, placental abruption was associated with the presence of a male fetus. One study stratified their analyses for parity (nulliparous vs multiparous). In both groups, placental abruption was associated with the presence of a male fetus.

In our pooled meta-analyses which compared the occurrence of placental abruption in women carrying a male fetus vs women carrying a female fetus, the OR was 1.07 (0.93–1.23) (Fig. 2h). The PAF for placental abruption was 1.18% (95% CI [1.05;2.46], *p* < 0.001). Assuming a worldwide prevalence of 1%, this resembles almost 38.000 cases of placental abruption worldwide associated so some degree with the presence of a male fetus [92].

#### Fetal sex and post-partum hemorrhage

Of the included studies, three investigated post-partum hemorrhage, with a total of 103.123 participants (Tables 1 and 2) [20, 42, 71]. One study found an association with the presence of a female fetus. This study however excluded preterm births. The other two studies did not find an association.

#### Fetal sex and miscarriage

Of the included studies, three investigated miscarriage, with a total of 1.217 participants (Tables 1 and 2) [72–74]. One study found an association between miscarriages and female sex. One other study stratified for morphological normal and abnormal embryos showing an association with male sex within the morphological normal embryos. The third study stratified their analyses for gestational age. In the total group and in the group 4–10 weeks, an association was found for female sex.

### Study quality, heterogeneity, and sensitivity analyses

Study quality according to the Newcastle-Ottawa scale was good. Over 90% of all included studies had a quality score of ≥ 6 out of 8 and 15% percent of studies had the maximum score of 8.

In a separate sensitivity analysis, all studies with specific in- or exclusion criteria were excluded for the meta-analyses. All results remained the same except for preterm pre-eclampsia, OR 0.85 (0.81–0.89). Furthermore, all analyses were stratified according to geographical location, number of participants, study design and quality score (Table 3). Stratified analysis for gestational hypertension by the level of quality score showed that only in the low-quality studies (i.e., quality score < 7) an association with male fetal sex was found (*p* < 0.001). For eclampsia, stratification by the number of participants showed no association with fetal sex in the larger studies (i.e., ≥ 10.000 participants) and an association with female fetal sex in one smaller study (*p* = 0.02). When stratifying by study design an association between female fetal sex and eclampsia was found in the one included case-control study. On the contrary, in the one included prospective cohort study an association with male fetal sex was found. In the five included retrospective cohort no association with fetal sex could be found (p = 0.01).

**Table 3:** Pooled odds ratios of the occurrence of maternal pregnancy complications by study characteristics

Subgroup	No. studies	Participants	OR (95% CI)	***p*** value for heterogeneity
**Gestational hypertension**				
Geographical location				
Western	12	5.511.490	1.02 (0.98;1.06)	0.29
Non-Western	5	125.016	0.99 (0.95;1.02)	
No of participants				
< 10.000	9	31.003	0,98 (0.86;1.10)	0.56
≥ 10.000	8	5.605.503	1,01 (0.98;1.05)	
Study design				
Case-control	2	444	0.86 (0.35;2,07)	0.57
Retrospective cohort	11	5.508.737	1.02 (0.98;1.05)	
Prospective cohort	4	127.325	0.98 (0.89;1.08)	
Quality score				
< 7	11	5.489.916	1.03 (1.01;1.05)	< 0.001
≥ 7	6	146.590	0.94 (0.82;1.06)	
**Pre-eclampsia (total)**				
Geographical location				
Western	22	3.970.495	1.02 (1.00;1.05)	0,23
Non-Western	15	636.671	0.93 (0.83;1,04)	
No of participants				
< 10.000	18	42.194	0.92 (0.82;1.04)	0.27
≥ 10.000	19	4.548.703	1,00 (0.96;1.03)	
Study design				
Case-control	9	18.593	0.94 (0.75;1.02)	0.50
Retrospective cohort	24	4.461.201	1,00 (0.96;1.04)	
Prospective cohort	4	127.372	0.90 (0.81;1.00)	
Quality score				
< 7	22	1.539.869	0.97 (0.93;1.02)	0.71
≥ 7	7	3.067.297	0.95 (0.88;1.02)	
**Eclampsia**				
Geographical location				
Western	5	4.820.821	1.02 (1.00;1.04)	0.05
Non-Western	2	110.156	0.82 (0.57;1.18)	
No of participants				
< 10.000	1	434	0.65 (0.45;0.94)	0.02
≥ 10.000	6	4.930.534	1.01 (0.99;1.04)	
Study design				
Case-control	1	434	0.65 (0.45;0.95)	0.01
Retrospective cohort	5	4.820.821	0.95 (0.88;1.02)	
Prospective cohort	1	109.722	1.02 (1.00;1.04)	
Quality score				
< 7	6	4.920.963	1.00 (0.95;1.04)	0.84
≥ 7	1	10.014	0.92 (0.42;2.01)	
**Gestational diabetes**				
Geographical location				
Western	18	1.728.325	1.03 (1.01;1.05)	0.13
Non-Western	10	380.388	1.07 (1.03;1.12)	
No of participants				
< 10.000	13	16.484	1.12 (1.02;1.24)	0.13
≥ 10.000	15	2.092.229	1.04 (1.02;1.06)	
Study design				
Case-control	6	1.092	1.15 (0.95;1.39)	0.66
Retrospective cohort	14	2.105.377	1.04 (1.02;1.06)	
Prospective cohort	8	2.246	1.16 (1.02;1.31)	
Quality score				
< 7	21	1.092.636	1.05 (1.02;1.09)	0.75
≥ 7	7	1.016.077	1.04 (1.02;1.06)	
**Placental abruption**				
Geographical location				
Western	7	2.876.604	1.03 (0.86;1.23)	0.45
Non-Western	6	227.068	1.10 (0.93;1.31)	
No of participants				
< 10.000	4	7.801	1.31 (0.85;2.02)	0.4
≥ 10.000	9	3.095.871	1.04 (0.90;1.22)	
Study design				
Case-control	2	1.090	2.34 (1.25;4.35)	0.08
Retrospective cohort	10	2.992.860	1.05 (0.90;1.22)	
Prospective cohort	1	109.722	0.98 (0.83;1.15)	
Quality score				
< 7	6	224.641	1.21 (0.96;1.51)	0.2
≥ 7	7	2.879.031	1.01 (0.85;1.19)	

Four of eight analyses showed high between-study heterogeneity, with an *I*^2^ estimate exceeding 75% (*p* < 0.05 for the Cochrane *X*^2^ statistic) (Fig. 2). This level of heterogeneity could be explained by differences between studies attributable to heterogeneous study populations, methods, and outcome definition.

## Discussion

This is the first systematic review and meta-analyses investigating the association between fetal sex and multiple major pregnancy outcomes showing that sexual dimorphisms in maternal pregnancy complications exist.

Within pre-eclampsia diverse results were found when stratifying for gestational age. Pregnancies with a female fetus were tended to be associated with preterm pre-eclampsia, while pregnancies with a male fetus were associated with developing term and postterm pre-eclampsia. This phenomenon is in line with results presented in a recent individual patient meta-analysis where women with a female fetus were more at risk for preterm pre-eclampsia and women with a male fetus for term pre-eclampsia [93]. In line with this, sexual dimorphic differences in vascular adaptation to pregnancy have been shown [9]. Women carrying a male fetus have a higher second-trimester uterine artery pulsatility index and more often present themselves with notching in the third trimester of pregnancy. This reflects an increased utero-placental resistance among male pregnancies which may originate from suboptimal implantation and placentation. A time diverse pattern was also seen in previous research on fetal sex-specific differences in blood pressure patterns during pregnancy [9]. Within complicated pregnancies (including pre-eclampsia) a different diastolic blood pressure was observed for women with a male fetus compared with women with a female fetus, with cross-over in the second trimester. Women carrying a female fetus started with a higher diastolic blood pressure compared with women carrying a male fetus. However, from 24 weeks of gestation onwards these women had a lower diastolic blood pressure. Although the exact underlying mechanisms of these changing patterns are still subject of investigation they might strengthen the hypothesis that pregnancies with a male embryo are more susceptible to suboptimal implantation or abnormal placental development which consequently leads to altered maternal adaptation to pregnancy. Recently Gonzalez et al. reported on the later first-trimester placental transcriptome [8]. They observed sexual dimorphic expression patterns of not only X- but also Y-linked genes in first-trimester placentas. Cell adhesion, ciliogenesis, and cell-cell communication genes also differed in their study. This suggests sex differences in how placenta cells interact with their environment [94–97]. Furthermore, they observed a significant downregulation of the ITGB8 gene (encodes integrin-β8). This gene promotes tumor angiogenesis and invasiveness in glioblastoma [97] functions necessary for normal first-trimester development when placental cells invade maternal tissue and access maternal blood. The results of Gonzalez et al. underscribe those of previous research by Cvitic et al. They found fetal sex differentially affected gene expression in a cell phenotype–dependent manner among cytotrophoblasts, syncytiotrophoblast, arterial and venous endothelial cells. The pathways that they observed in male placenta villi were identified to be signaling pathways for graft-versus-host disease as well as the immune and inflammatory systems that parallel the reported poorer outcome of male fetuses [98]. Orzack et al. studied the trajectory of the human sex ratio from conception to birth by analyzing data from 3 to 6 days old embryos, including abortions, chorionic villus sampling, amniocentesis, fetal deaths, and live births. They showed a sex ratio among abnormal embryos that was male biased, and a sex ratio among normal embryos that was female biased. This strengthened the study of Buckberry et al. who detected a higher female expression from genes involved in the maintenance of pregnancy and the maternal immune tolerance of the conceptus [6]. From this, we, and others, speculate that pregnancies with a male embryo are more susceptible to impaired placentation. This would imply that those pregnancies with a male embryo that are susceptible to develop pre-eclampsia due to impaired placentation may already have miscarried in the first trimester [98–100]. The male fetuses that survive the period of placentation will thereby represent a relatively healthy group of fetuses leading to a female-biased prevalence of pre-eclampsia [99]. Since especially late-onset pre-eclampsia is thought to originate from abnormal placentation a so-called sexual dimorphic cross-over can be observed for term and postterm pre-eclampsia [4, 6, 7, 72, 98, 100].

The implication that male embryos are more susceptible to placental development is in line with the results described in this systematic review since other placental related pregnancy complications are also mainly associated with the presence of a male fetus. Although beyond the scope of this review, this is in line with the association of the presence of a male fetus with preterm birth [101]. Many cases of spontaneous preterm birth appear to be caused by placental insufficiency, similar to pre-eclampsia. Other causes of preterm birth including placental abruption and chronic villitis also have specific placental pathology related to placental insufficiency and are also associated with male sex [102]. Furthermore, we hypothesize that carrying a male fetus demands a higher degree of metabolic and vascular maternal adaptation to pregnancy compared with carrying a female fetus. For example, women carrying a male fetus have poorer pancreatic beta-cell function in pregnancy [64]. This is in line with our finding that women carrying a male fetus are at higher risk for developing gestational diabetes. Previous research also showed that within women who experienced gestational diabetes, those women who carried a male fetus are at higher risk of developing diabetes type 2 after delivery compared with women who carried a female fetus [85].

Not only during pregnancy the consequences of carrying a male fetus for maternal health are evident. Also, long term adverse health outcomes have been measured. Helle et al. were the first to suggest a shorter maternal lifespan is associated with the number of sons born [103]. More recently research has shown that that women’s post-reproductive survival declines with the number of sons they gave birth to [104, 105]. The number of daughters born was not associated with women’s post-reproductive survival. Helle et al. validated their results by demonstrating that this effect was independent on the number of sons and daughters surviving to adulthood and by showing that the number of sons and daughters was not associated with post-reproductive survival in men [104]. These findings support the hypothesis that baring sons is more energetically costly than baring daughters.

Conclusions on fetal sex and miscarriage rates are difficult to draw from the included studies. One of our exclusion criteria was an abnormal karyogram, which is highly prevalent in miscarriages [106]. This could have introduced a selection bias if an abnormal karyogram occurs more often in male pregnancies and give rise to a female dominance in miscarriages with a normal karyogram while in the total group of miscarriages there is a male dominance. Furthermore, the pregnancy product after a miscarriage is only investigated in specific cases like recurrent miscarriages and is not part of daily practice. To investigate if a sexual dimorphism in miscarriages exists, future research should focus on the total rate of miscarriages, stratified for chromosomal abnormalities.

To our knowledge, this is the first comprehensive quantitative review that assessed the association between fetal sex and multiple major pregnancy outcomes. Our analyses included over 12 million women and assessed seven pregnancy outcomes. Some systematic reviews exist focusing on one pregnancy complication, for example on gestational diabetes and pre-eclampsia/eclampsia by Jaskolka et al. [107, 108]. Eleven of our 25 included studies on gestational diabetes and 22 of our 31 included studies on pre-eclampsia were not included in these systematic reviews. For pre-eclampsia, this resulted in one million more participants included. In the systematic review and meta-analyses on pre-eclampsia, the authors unfortunately do not take into account that the effect of fetal sex on the occurrence of pre-eclampsia is gestational age specific and therefore stratification into preterm, term and postterm pre-eclampsia was not performed.

However, strength and limitations in the current study merit careful consideration. First, all systematic reviews are prone to reporting bias, owing to the possibility that studies with more extreme results are more likely to be published. In this systematic review, multiple included articles did not primarily investigate the effect of fetal sex on pregnancy outcome. However, due to the fact that the information was given anyway in the manuscript, odds ratios could be calculated. Additionally, all meta-analyses are limited by the quality of the individual published studies. However, the majority of studies included in the current analyses were of high quality, with a low risk of bias. Furthermore, the majority of studies did not give a clear definition of the pregnancy outcome which was assessed. Also, definition changed internationally across time. The publication year of included studies varies between 1970 and 2019. In this time span, the definition of several pregnancy complications such as pre-eclampsia and gestational diabetes have changed multiple times. Moreover, there might not be international consensus to a definition which causes other definition in different continents or countries. This introduces heterogeneity into the analyses.

Most studies that were included did not adjust for any confounders. From an epidemiological point of view, when using fetal sex as an exposure we don’t have to deal with any confounding factors since there are no factors described influencing fetal sex.

## Conclusions

Our findings support the emerging concept of a sexual dimorphism in the maternal-fetal-placental interplay. Most importantly all results are consistent with each other and validate the hypothesis that carrying a male fetus is accompanied with a higher cardiovascular and metabolic load for the mother resulting in maternal pregnancy complications and adverse health in later life. Although the increases in odds ratios in this meta-analysis are modest, they hold important implications for our understanding of maternal-fetal physiology. Moreover, approximately half of pregnant women worldwide are exposed to the presence of a male fetus. Hence, the absolute numbers of pregnancy complications worldwide occurring due to the presence of a male fetus are high. Experiencing one of the pregnancy complications described in this systematic review holds important implications for future life. Fetal sex should therefore be taken into account as a risk factor when assessing risk of pregnancy complications and adverse cardiovascular health in later life.

## Supplementary information

**Additional file 1.** Search Strategy.

**Additional file 2.** Newcastle-Ottawa Quality Assessment Scale.

**Additional file 3.** PRISMA checklist.

**Additional file 4.** MOOSE checklist.

## Data Availability

The extracted data from included articles supporting the conclusions of this manuscript can be found in Table 1 and 2.
