# Supplementary material for: Fetal sex and maternal pregnancy outcomes: a systematic review and meta-analysis
Source: Biol Sex Differ. 2020 May 11;11:26. doi: 10.1186/s13293-020-00299-3 (PMC7216628; doi:10.1186/s13293-020-00299-3)
Supplement: Supplementary file 1 — Additional file 1. Search Strategy. [file 13293_2020_299_MOESM1_ESM.docx]

**Additional file 1** Search Strategy

Relevant articles, published before August 19^th^ 2015, were identified through six electronic databases (Embase (in embase.com), Medline (in OvidSP), Cochrane central (in Wiley), Web-of-Science, Pubmed as supplied by publisher and Google Scholar). The following search terms were used without language restriction:

**Embase.com**

(((gender/de OR 'sex difference'/exp) AND fetus/exp) OR (((gender* OR femal* OR male* OR sex OR boy* OR girl*) NEAR/6 (fetus* OR fetal OR foetus* OR foetal OR embryo* OR baby OR babies))):ab,ti) AND ('pregnancy outcome'/exp OR 'pregnancy disorder'/exp OR 'pregnancy complication'/exp OR 'Doppler flowmetry'/exp OR 'Doppler flowmeter'/exp OR 'birth weight'/exp OR 'intrauterine growth retardation'/exp OR 'prenatal growth'/exp OR mortality/de OR 'fetus mortality'/de OR 'embryo mortality'/de OR 'perinatal mortality'/exp OR 'prenatal mortality'/exp OR (((pregnan* OR gravid* OR gestation* OR maternal* OR mother* OR intrauterin* OR neonat* OR newborn* OR prenatal*) NEAR/6 (mortalit* OR surviv* OR fatal* OR outcome* OR growth* OR disorder* OR disease* OR complication* OR toxemi* OR hypertens* OR 'blood pressure' OR small* OR large* OR duration OR prolong*)) OR eclamp* OR preeclamp* OR Doppler OR notching OR ((birth ) NEAR/3 (weight OR length*)) OR birthweight OR lbw OR elbw OR vlbw OR iugr OR fgr OR sga OR macrosomi* OR stillbirth OR stillborn OR (live NEXT/1 birth*) OR prematur* OR postmatur* OR imematur* OR preterm OR postterm OR dysmatur* OR serotinit* OR miscarriage* OR mortalit* OR (spontaneous NEAR/3 abortion*)):ab,ti) NOT ([animals]/lim NOT [humans]/lim) NOT ([Conference Abstract]/lim OR [Letter]/lim OR [Note]/lim OR [Conference Paper]/lim OR [Editorial]/lim OR 'case report'/exp OR 'case report':ti) AND [english]/lim

**Medline (OvidSP)**

((sex/ AND fetus/) OR (((gender* OR femal* OR male* OR sex OR boy* OR girl*) ADJ6 (fetus* OR fetal OR foetus* OR foetal OR embryo* OR baby OR babies))).ab,ti.) AND ("Fetal Mortality"/ OR "Maternal Mortality"/ OR "Perinatal Mortality"/ OR exp pregnancy outcome/ OR pregnancy complications/ OR exp Ultrasonography, Doppler/ OR birth weight/ OR exp Infant, Low Birth Weight/ OR Fetal Growth Retardation/ OR (((pregnan* OR gravid* OR gestation* OR maternal* OR mother* OR intrauterin* OR neonat* OR newborn* OR prenatal*) ADJ6 (mortalit* OR surviv* OR fatal* OR outcome* OR growth* OR disorder* OR disease* OR complication* OR toxemi* OR hypertens* OR "blood pressure" OR small* OR large* OR duration OR prolong*)) OR eclamp* OR preeclamp* OR Doppler OR notching OR ((birth ) ADJ3 (weight OR length*)) OR birthweight OR lbw OR elbw OR vlbw OR iugr OR fgr OR sga OR macrosomi* OR stillbirth OR stillborn OR (live ADJ birth*) OR prematur* OR postmatur* OR imematur* OR preterm OR postterm OR dysmatur* OR serotinit* OR miscarriage* OR mortalit* OR (spontaneous ADJ3 abortion*)).ab,ti.) NOT ((letter OR news OR comment OR editorial OR congresses OR abstracts OR case reports).pt. OR case report.ti.) AND english.la.

**Cochrane central**

((((gender* OR femal* OR male* OR sex OR boy* OR girl*) NEAR/6 (fetus* OR fetal OR foetus* OR foetal OR embryo* OR baby OR babies))):ab,ti) AND ((((pregnan* OR gravid* OR gestation* OR maternal* OR mother* OR intrauterin* OR neonat* OR newborn* OR prenatal*) NEAR/6 (mortalit* OR surviv* OR fatal* OR outcome* OR growth* OR disorder* OR disease* OR complication* OR toxemi* OR hypertens* OR 'blood pressure' OR small* OR large* OR duration OR prolong*)) OR eclamp* OR preeclamp* OR Doppler OR notching OR ((birth ) NEAR/3 (weight OR length*)) OR birthweight OR lbw OR elbw OR vlbw OR iugr OR fgr OR sga OR macrosomi* OR stillbirth OR stillborn OR (live NEXT/1 birth*) OR prematur* OR postmatur* OR imematur* OR preterm OR postterm OR dysmatur* OR serotinit* OR miscarriage* OR mortalit* OR (spontaneous NEAR/3 abortion*)):ab,ti)

**Web-of-science**

TS=(((((gender* OR femal* OR male* OR sex OR boy* OR girl*) NEAR/5 (fetus* OR fetal OR foetus* OR foetal OR embryo* OR baby OR babies)))) AND ((((pregnan* OR gravid* OR gestation* OR maternal* OR mother* OR intrauterin* OR neonat* OR newborn* OR prenatal*) NEAR/5 (mortalit* OR surviv* OR fatal* OR outcome* OR growth* OR disorder* OR disease* OR complication* OR toxemi* OR hypertens* OR "blood pressure" OR small* OR large* OR duration OR prolong*)) OR eclamp* OR preeclamp* OR Doppler OR notching OR ((birth ) NEAR/2 (weight OR length*)) OR birthweight OR lbw OR elbw OR vlbw OR iugr OR fgr OR sga OR macrosomi* OR stillbirth OR stillborn OR (live NEAR/1 birth*) OR prematur* OR postmatur* OR imematur* OR preterm OR postterm OR dysmatur* OR serotinit* OR miscarriage* OR mortalit* OR (spontaneous NEAR/2 abortion*))) NOT ((animal* OR mouse* OR mice OR rat OR rats OR murine OR bovine OR ovine OR porcine OR cow OR pig OR sheep OR rabbit*) NOT (human* OR patient* OR woman* OR women* OR baby OR babies))) AND DT=(Article) AND LA=(english)

**PubMed publisher as supplied by publisher**

((sex[mh] AND fetus[mh]) OR Female fetus*[tiab] OR female embryo*[tiab] OR male fetus*[tiab] OR male embryo*[tiab] OR fetal gender*[tiab] OR embroynal gender*[tiab] OR fetal sex*[tiab] OR embroynal sex*[tiab]) AND ("Fetal Mortality"[mh] OR "Maternal Mortality"[mh] OR "Perinatal Mortality"[mh] OR pregnancy outcome[mh] OR pregnancy complications[mh] OR Ultrasonography, Doppler[mh] OR birth weight[mh] OR Infant, Low Birth Weight[mh] OR Fetal Growth Retardation[mh] OR (((pregnan*[tiab] OR gravid*[tiab] OR gestation*[tiab] OR maternal*[tiab] OR mother*[tiab] OR intrauterin*[tiab] OR neonat*[tiab] OR newborn*[tiab] OR prenatal*[tiab]) AND (mortalit*[tiab] OR surviv*[tiab] OR fatal*[tiab] OR outcome*[tiab] OR growth*[tiab] OR disorder*[tiab] OR disease*[tiab] OR complication*[tiab] OR toxemi*[tiab] OR hypertens*[tiab] OR "blood pressure" OR small*[tiab] OR large*[tiab] OR duration OR prolong*[tiab])) OR eclamp*[tiab] OR preeclamp*[tiab] OR Doppler OR notching OR ((birth ) AND (weight OR length*[tiab])) OR birthweight OR lbw OR elbw OR vlbw OR iugr OR fgr OR sga OR macrosomi*[tiab] OR stillbirth OR stillborn OR (live ADJ birth*[tiab]) OR prematur*[tiab] OR postmatur*[tiab] OR imematur*[tiab] OR preterm OR postterm OR dysmatur*[tiab] OR serotinit*[tiab] OR miscarriage*[tiab] OR mortalit*[tiab] OR (spontaneous AND abortion*[tiab]))) NOT ((letter[pt] OR news[pt] OR comment[pt] OR editorial[pt] OR congresses[pt] OR abstracts[pt] OR case reports[pt]) OR case report[ti]) AND english[la] AND (publisher[sb] OR inprocess [sb])

**Google Scholar**

"female|male fetus|fetal|embryo"|"fetal|embryonal gender|sex" "pregnancy|fetus|fetal|maternal|neonatal|newborn mortality |survival|outcome|growth|disorders|diseases|complications"|stillbirth
