# Supplementary material for: Fetal sex and maternal pregnancy outcomes: a systematic review and meta-analysis
Source: Biol Sex Differ. 2020 May 11;11:26. doi: 10.1186/s13293-020-00299-3 (PMC7216628; doi:10.1186/s13293-020-00299-3)
Supplement: Supplementary file 2 — Additional file 2. Newcastle-Ottawa Quality Assessment Scale. [file 13293_2020_299_MOESM2_ESM.docx]

**Additional file 2** Newcastle-Ottawa Quality Assessment Scale

**Case control studies**

Note: A study can be awarded a maximum of one star for each numbered item within the Selection and Exposure categories. A maximum of two stars can be given for Comparability.

**Selection**

1. Is the case definition adequate
2. Yes, with independent validation *
3. Yes, eg record linkage or based on self reports
4. No description
5. Representativeness of the cases
6. Consecutive or obviously representative series of cases *
7. Potential for selection biases or not stated
8. Selection of Controls
9. Community records *
10. Hospital controls
11. No description
12. Definition of Controls
13. No history of disease (endpoint) *
14. No description of source

**Comparability**

1. Comparability of cases and controls on the basis of the design or analyses
2. Study controls for … (Select the most important factor) *
3. Study controls for any additional factor * (This criteria could be modified to indicate specific control for a second important factor)

**Exposure**

1. Ascertainment of exposure
2. Secure record (eg surgical records) *
3. Structured interview where blind to case/control status *
4. Interview not blinded to case/control status
5. Written self report or medical record only
6. No description
7. Same method of ascertainment for cases and controls
8. Yes *
9. No
10. Non-Response rate
11. Same rate for both groups *
12. Non respondents described
13. Rate different and no designation

**Cohort studies**

Note: A study can be awarded a maximum of one star for each numbered item within the Selection and Exposure categories. A maximum of two starts can be given for Comparability.

**Selection**

1. Representativeness of the exposed cohort
2. Truly representative of the average … (describe) in the community *
3. Somewhat representative of the average … in the community *
4. Selected group of users eg nurses, volunteers
5. No description of the derivation of the cohort
6. Selection of the non exposed cohort
7. Drawn from the same community as the exposed cohort *
8. Drawn from a different source
9. No description of the derivation of the non exposed cohort
10. Ascertainment of exposure
11. Secure record (eg surgical records) *
12. Structured interview *
13. Written self report
14. No description
15. Demonstration that outcome of interest was not present at start of study
16. Yes *
17. No

**Comparability**

1. Comparability of cohorts on the basis of the design or analysis
2. Study controls for … (select the most important factor) *
3. Study controls for any additional factor * (This criteria could be modified to indicate specific control for a second important factor)

**Outcome**

1. Assessment of outcome
2. Independent blind assessment *
3. Record linkage *
4. Self report
5. No description
6. Was follow-up long enough for outcome to occur
7. Yes (select an adequate follow up period for outcome of interest) *
8. No
9. Adequacy of follow up of cohorts
10. Complete follow up – all subjects accounted or *
11. Subjects lost to follow up unlikely to introduce bias – small number lost -> … % (select and adequate %) follow up, or description provided of those lost) *
12. Follow up rate < … % (select an adequate %) and no description of those lost
13. No statement
