# Supplementary material for: Fetal sex and maternal pregnancy outcomes: a systematic review and meta-analysis
Source: Biol Sex Differ. 2020 May 11;11:26. doi: 10.1186/s13293-020-00299-3 (PMC7216628; doi:10.1186/s13293-020-00299-3)
Supplement: Supplementary file 4 — Additional file 4. MOOSE checklist. [file 13293_2020_299_MOESM4_ESM.docx]

**Additional file 4:** MOOSE checklist

| **Criteria** | | **Brief description of how the criteria were handled in the meta-analysis** |
| --- | --- | --- |
| **Reporting of background should include** | |  |
| √ | Problem definition | Multiple maternal pregnancy complications are placenta mediated. Since the placenta also has a sex, fetal sex-specific differences in the occurrence of these complications could exist. A meta-analysis on multiple pregnancy complications has not been performed previously. |
| √ | Hypothesis statement | Male fetal sex is associated with multiple maternal pregnancy complications. |
| √ | Description of study outcomes | We included studies on multiple maternal pregnancy complications, including: gestational hypertension, pre-eclampsia, eclampsia, gestational diabetes, placental abruption, post-partum haemorrhage and miscarriage. |
| √ | Type of exposure or intervention used | Fetal sex. |
| √ | Type of study designs used | All observational study designs including cohort and case-controls. |
| √ | Study population | Only studies carried out in singleton pregnancies were included. Studies on newborns with an abnormal karyogram, congenital conditions involving sex steroids and/or sex characteristics were excluded. |
| **Reporting of search strategy should include** | |  |
| √ | Qualifications of searchers | The credentials of the investigators are indicated in the authors list. |
| √ | Search strategy, including time period included in the synthesis and keywords | Search strategy and time periods are detailed in page 5 of the manuscript and in Figure 1. |
| √ | Databases and registries searched | Ovid MEDLINE, EMBASE, Cochrane Central, Web-of-Science, Pubmed and Google Scholar. |
| √ | Search software used, name and version, including special features | We did not employ a search software. Endnote was used to merge retrieved citations and eliminate duplications |
| √ | Use of hand searching | We hand-searched bibliographies of retrieved systematic reviews and meta-analysis for additional references. |
| √ | List of citations located and those excluded, including justifications | Details of the literature search process are outlined in the flow chart. The citation list for excluded studies is available upon request. |
| √ | Method of addressing articles published in languages other than English | We placed no restrictions on language; local translation services were available |
| √ | Method of handling abstracts and unpublished studies | Systematic reviews were used to identify further references. Authors of included studies were contacted to retrieve missing full texts and to identify any missing studies. |
| √ | Description of any contact with authors | Authors of included studies were contacted to retrieve missing full texts and to identify any missing studies. |
| **Reporting of methods should include** | |  |
| √ | Description of relevance or appropriateness of studies assembled for assessing the hypothesis to be tested | Detailed inclusion and exclusion criteria were described in the methods section. |
| √ | Rationale for the selection and coding of data | A predesigned data collection form was prepared to extract the relevant information from the included full texts, including study design, characteristics of the study participants and information on the reported outcome. |
| √ | Assessment of confounding | We performed qualitative analyses to evaluate differences between studies |
| √ | Assessment of study quality, including blinding of quality assessors; stratification or regression on possible predictors of study results | We used the Newcastle- Ottawa Scale (NOS) to evaluate the quality of case-control and cohort studies included in this review |
| √ | Assessment of heterogeneity | We were not able to pool due to large levels of heterogeneity evaluated visually and statistically |
| √ | Description of statistical methods in sufficient detail to be replicated | We conducted qualitative analysis of the data with sensitivity analyses and stratification on multiple variables. |
| √ | Provision of appropriate tables and graphics | We included 2 main figures, 1 main table, and 7 appendices |
| **Reporting of results should include** | |  |
| √ | Graph summarizing individual study estimates and overall estimate | Figure 1 |
| √ | Table giving descriptive information for each study included | In appendices. |
| √ | Results of sensitivity testing | The results on sensitivity analyses are described on page 12 and 13. |
| √ | Indication of statistical uncertainty of findings | 95% confidence intervals were presented if available |
| **Reporting of discussion should include** | |  |
| √ | Quantitative assessment of bias | Not applicable |
| √ | Justification for exclusion | We excluded studies that had no or unclear definition of exposure and outcome, or data extraction was not feasible. Studies on newborns with an abnormal karyogram, congenital conditions involving sex steroids and/or sex characteristics were excluded. |
| √ | Assessment of quality of included studies | We used the Newcastle- Ottawa Scale (NOS) to evaluate the quality of case-control and cohort studies included in this review. |
| **Reporting of conclusions should include** | |  |
| √ | Consideration of alternative explanations for observed results | Since fetal sex is random and non-confounded alternative explanations for the observed results are unlikely. |
| √ | Generalization of the conclusions | The generalizability of our findings has been enhanced by the involvement of data from multiple regions in the world, including North-America, Europe, the Middle-East, Australia, Asia and Africa. However there is a clear lack of evidence from South-America. |
| √ | Guidelines for future research | Further work is necessary to elucidate the pathophysiological background of the observed sexual dimorphism in maternal-fetal-interplay. |
| √ | Disclosure of funding source | Completion of this manuscript was supported without any funding. |
